# Deep learning techniques for isointense infant brain tissue segmentation: a systematic literature review

**DOI:** 10.3389/fmed.2023.1240360

**Published:** 2023-12-18

**Authors:** Sandile Thamie Mhlanga, Serestina Viriri

**Affiliations:** School of Mathematics, Statistics and Computer Science, University of KwaZulu-Natal, Durban, South Africa

**Keywords:** isointense infant brain, segmentation, deep learning, convolutional neural networks, magnetic resonance imaging

## Abstract

**Introduction:**

To improve comprehension of initial brain growth in wellness along with sickness, it is essential to precisely segment child brain magnetic resonance imaging (MRI) into white matter (WM) and gray matter (GM), along with cerebrospinal fluid (CSF). Nonetheless, in the isointense phase (6-8 months of age), the inborn myelination and development activities, WM along with GM display alike stages of intensity in both T1-weighted and T2-weighted MRI, making tissue segmentation extremely difficult.

**Methods:**

The comprehensive review of studies related to isointense brain MRI segmentation approaches is highlighted in this publication. The main aim and contribution of this study is to aid researchers by providing a thorough review to make their search for isointense brain MRI segmentation easier. The systematic literature review is performed from four points of reference: (1) review of studies concerning isointense brain MRI segmentation; (2) research contribution and future works and limitations; (3) frequently applied evaluation metrics and datasets; (4) findings of this studies.

**Results and discussion:**

The systemic review is performed on studies that were published in the period of 2012 to 2022. A total of 19 primary studies of isointense brain MRI segmentation were selected to report the research question stated in this review.

## 1 Introduction

In brain research, the precise separation of infant brain tissues into non-overlapping regions such as white matter (WM), gray matter (GM) and cerebrospinal fluid (CSF) is crucial for determining how the normal and abnormal development of the developing brain (1–3). The first year of life is the most dynamic period in the development of the human brain, with fast tissue growth and the emergence of a vast array of cognitive and physical abilities (4, 5). Major brain diseases that are difficult to treat, such as attention deficit hyperactivity disorder (ADHD), baby autism, bipolar affective disorder, and schizophrenia, may show up in the patient’s developing brain tissue (6). Therefore, it is important that brain structures are adequately segmented in new-born images. The aim of precise brain tissue image segmentation is to provide crucial information for clinical diagnostics, treatment assessments, analysing brain changes, enabling clinical preparations together with presenting image-guided interventions (7–9).

Thus far, magnetic resonance imaging (MRI) is the predominant technique for imaging baby brain, specifically T1-weighted and T2-weight MRI, because it is safe, non-invasive and attains non-intrusive cross-sectional views of the brain in multiple contrast without ionizing radiation (10, 11). Compared to automated segmentation, manual segmentation is tremendously arduous and time-consuming assignment which compels a comprehensive expertise base of brain structure and impossible at large scale. In addition, manual segmentation experiences small reproducibility, which is highly inclined to errors due to inter or inter-operator unpredictability (7, 8, 12, 13). Therefore, precise and automatic segmentation methods are highly needed.

**Figure 1:**
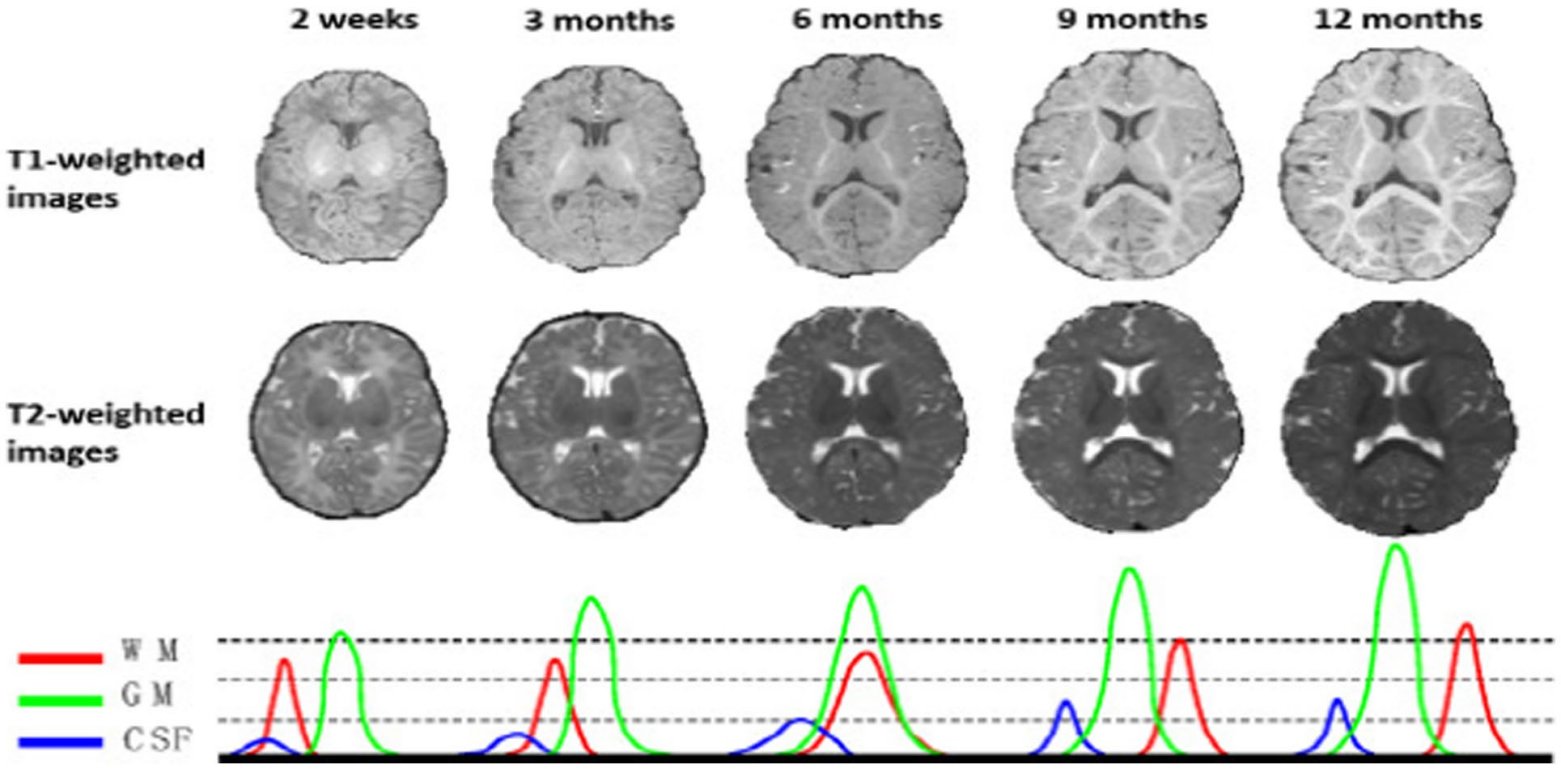
T1 and T2 weighted MRI images of a baby taken at various ages—2 weeks, 3, 6, 9, and 12 months. The MR images of infants around 6 months old (i.e., the isointense phase) show the lowest tissue contrast, indicating the most difficult tissue segmentation. The bottom row displays the equivalent tissue intensity distributions from T1w MR images, where the WM and GM intensities are heavily overlapping during the isointense period. Reprinted with permission from IEEE, Copyright © 2019 IEEE (16).

Infant brain MRI segmentation is recognized to be far more challenging than adult brain segmentation (5), due to ongoing white matter myelination, significant partial volume effects, decreased tissue contrast (14), increased noise, and infant brain pictures (14, 15). In actuality, as depicted in Figure 1, there are three distinct phases in the first-year brain MRI (16). Gray matter exhibits a higher signal strength than white matter in T1-weighted images during (1) the infancy phase (5 months). The gray matter has the lowest signal differentiation with the white matter in both T1 and T2 imaging during the second isointense phase (6–9 months), in which the signal intensity of white matter is growing during development due to myelination and maturation process. The final stage is the early adult-like stage (9 months), where the distribution of gray matter intensity in T1 images is significantly lower than that of white matter, resembling the pattern of tissue contrast in adult MRI (5, 16).

Furthermore, the intensity distributions of the voxels in the gray and white matter continue to heavily overlap in the isointense stage, particularly in the cortical areas, in this way driving to the least tissue differentiation and making the primary challenging for tissue segmentation, in relationship to pictures on previous stages of brain development (5, 16–18). Numerous efforts have been made in the past few years to segment the baby brain using MRI (4, 6, 11, 19–28).

Despite having an array of infant brain segmentation models, to determine which segmentation techniques are most frequently employed and in what combinations, there is a need to assess the body of literature as a whole using a systematic literature review paper. By doing this, the restrictions on personal searches for isointense brain MRI segmentation models would be lessened. What are the current isointense brain MRI segmentation algorithms, and what are the application challenges? Is the main research question leading this systematic literature review (SLR). As a result, the study’s goal is to examine isointense brain MRI segmentation models utilizing a literature review.

## 2 Literature review

As of late, deep learning techniques centred around convolutional neural networks (CNNs) have demonstrated exceptional execution around a range of computer visualization and photograph evaluation usages in the clinical space (16, 17, 29–32). CNNs have accomplished advanced outcomes in numerous brain segmentation tribulations (7, 8, 12, 33–36), including the subdivision of 6-moths old brain MRI (1, 11, 21, 22, 24, 25, 32, 37, 38).

Some researchers have refined many recognized CNNs, for example U-Net (36, 38, 39) and the DenseNet (11, 21, 24, 34), for brain MRI division on 6-months-old child (1, 40, 41). These methods improve the viable conveyance and combination of the semantic data in a multimodal characteristics and have accomplished enhanced functioning contrasted with common machine learning techniques (16, 17). Nevertheless, inadequacies however occur in the present CNN-based division techniques for child brain for example, previous models focus on enhancing network architecture for example modality blend (41) and interlayer links (37, 42), which requires seasoned expertise experience for network designing and the training turn out to be more challenging as the network amplifies the depth (21). Furthermore, hardware requirements for computing and memory escalates drastically as the depth increase (21). Combination of these methods for improved performance is also problematic due to the inconsistence network layouts, tedious hyper-parameter alteration and extreme graphic processing unit (GPU) memory utilization (17).

## 3 Methodology

The process of finding and critically evaluating pertinent research, as well as gathering and analysing data from this research, is known as a systematic literature review, or SRL (43). A systematic review’s objective is to locate all empirical data that satisfies the inclusion criteria and provides an answer to a particular research question (43, 44). Additionally, it takes time to separate the known from the unknown. That is a crucial justification for conducting SLRs in accordance with a set of clear-cut methodological stages (45). This study established a systematic literature review (SLR) on the segmentation of isointense brain MRI using the Preferred Reporting Items for Systematic Reviews and Meta-analyses (PRISMA). PRISMA is a well-known systematic review methodology that has been used in a variety of research domains, including the medical field (46), business (47) and safety mining (45). Because of its 27 evidence-based checklist and four-phase analysis, PRISMA is acceptable in the research area even if it is not a quality assessment approach. This allows systematic literature reviews (SLRs) to be clear and transparent (43, 48). Identification, screening, eligibility, and data abstraction and analysis are the four core PRISMA phases. This systematic review was conducted from 1 August 2022 to 31 December 2022.

### 3.1 Research question

This study assesses segmentation results of isointense brain MRI studies that have been conducted in the past. For the purpose of describing the systematic literature review, the following four research questions have been developed.

- [RQ-1] What techniques have been used for isointense brain MRI segmentation in neurosciences?
- [RQ-1a] What are the existing isointense brain MRI segmentation machine learning algorithm?
- [RQ-1b] What evaluation metrics have been used to measure accuracy of the techniques?
- [RQ-2] What are the characteristic of the dataset used in neurosciences for isointense brain MRI segmentation?
- [RQ-3] What are the findings of isointense brain MRI segmentation in this study?
- [RQ-4] What are the future works and limitations to ease the other researchers search for isointense brain MRI segmentation?

### 3.2 PRISMA phases

#### 3.2.1 Identification

The identification stage is the first step in the systematic literature review (SLR) process. The study question and goals are clearly defined at this point. A widespread search study was executed using Web of Science (WoS) and Scopus. All significant publishers, including Science Direct, Emerald, Taylor & Francis, Springer Links, IEEE, and Willey, are included in the Scopus integrated database. Due to its high calibre indexing information, many academics have regarded the Scopus database as a trustworthy resource for SLR. All appropriate peer-reviewed articles published between 2012 and December 31, 2022, are included in the search. When looking for pertinent publications, use terms like “*automatic isointense MRI brain segmentation*,” “*Image segmentation 6-month brain MRI*,” “Infant *brain tissue segmentation*,” and “*Segmentation neonatal brain MRI*.” The Boolean operators are combined with various keywords to enlarge the search range 634 articles were obtained as a consequence of this method from the combined Scopus and Web of Science databases (Table 1).

**Table 1:** Keywords used in this research.

Automatic Image Segmentation construct	AND	Group of participants’ construct	OR	Characteristic of interest construct
“Automatic segmentation” OR		“Isointense” OR		“brain MRI” OR
“Image segmentation” OR		“6-months” OR		“brain MRI tissues” OR
“Brain tissue segmentation” OR		“Infant” OR		“white matter (WM), gray matter (GM), and cerebrospinal fluid (CSF)” OR
“Segmentation”		“Neonatal” OR		“MRI brain tissues”

#### 3.2.2 Screening

The subsequent stage is the screening procedure, in which articles are included or excluded based on standards set by the writers. Tables 2–4 provide specifics regarding inclusion and exclusion. Following the identifying procedure, 634 articles needed to be screened. Duplications were identified and removed, and 580 for the title and abstract screening, articles were found. Relevant articles were forwarded to the candidate data. After reviewing all available literature, the candidate data set was reviewed, and the inclusion and exclusion criteria were used to populate the chosen data. The screening stage produced 167 publications that were only focused on isointense brain MRI segmentation and were published between January 2012 and December 31, 2022. Journals that published systematic reviews, review papers, proceedings from conferences, book chapters, book series, and novels were not included. The goal is to concentrate on legitimate isointense brain MRI segmentation research.

**Table 2:** Literature inclusion criteria.

Number	Criteria	Inclusion
1	Primary Source	Literature describes data collected and analysed by the authors and not based on the other research conclusion
2	Relevant topic	Literature directly references isointense infant brain image segmentation and provide analysis of the proposed models and the metrics used to evaluate the models
3	Publication timeline	January 2012 – December 2022
4	Review quality	Literature is published in a peer-reviewed journal
5	Dataset used	Studies that use iSeg-2017 and iSeg-2019 dataset.
6	Data quality	Literature must show data sources are numerous enough, qualified enough and representative enough to avoid bias in qualitative literature.

**Table 3:** Literature exclusion criteria.

Number	Criteria	Exclusion
1	Secondary Source	Article is a secondary source. Secondary data can distort this analysis by presenting a single model with multiple results.
2	Irrelevant studies	Literature that does not reference infant brain image segmentation, specifically isointense (6–8 months)
3	Publication timeline	2011 and before
4	Document type	Journals (systematic review), review papers, conference proceedings, dissertations, these, white papers, incomplete bibliographic records, industry reports, others on the basis of relevance, chapters in a book, book series, books
5.1	Unavailability	Literature is not available as a full-text article in the selected data source.
5.2		Literature not available in research data source at the time of data collection.
6.1	Inadmissible quality	Literature is not published in a peer-reviewed journal.
6.2		Literature does not adequately or completely its methodology such that it cannot determined how the model was created and evaluated.
6.3		Literature were T1-weighted and T2-weight MRI are not used.
6.4		Literature were fetal MRI imaged was used. (0–5 months).
6.5		Literature were not all 3 tissues (WM, GM and CSF) are segmented.
7	Language	Literature is not in English
8	Duplication	Literature is a duplicate of other literature in the study.

**Table 4:** Quality assessment checklist adopted from Kitchenham et al. (49) as cited by Usman et al. (50).

NO#	Question	Score
1	Are the research aim clearly specified?	Y|N|P
2	Was the study designed to achieve these aims?	Y|N|P
3	Are the segmentation techniques clearly described?	Y|N|P
4	Are the evaluation metrics used adequately described	Y|N|P
5	Are all research question answered adequately?	Y|N|P
6	Are negative (if any) presented?	Y|N|P
7	Are datasets considered by the study?	Y|N|P
8	Is the purpose of data analysis clear?	Y|N|P
9	Do the researcher discuss any problems with validity/reliability of the results	Y|N|P
10	How clear are the links between data interpretation and conclusion?	Y|N|P
11	Are finding based on multiple projects	Y|N|P
12	Are statistical techniques are used to analyse data adequately?	Y|N|P
13	Are data collection method adequately described?	Y|N|P

#### 3.2.3 Eligibility

The third phase is the eligibility procedure, in which articles are included or eliminated according to the precise standards set forth by the writers. Manual screening of literature with a focus on the segmentation of isointense brain MRI and the inclusion and exclusion criteria from previous screening processes. The review was able to collect 19 carefully chosen articles on isointense brain MRI segmentation.

#### 3.2.4 Data abstraction and analysis

**Figure 2:**
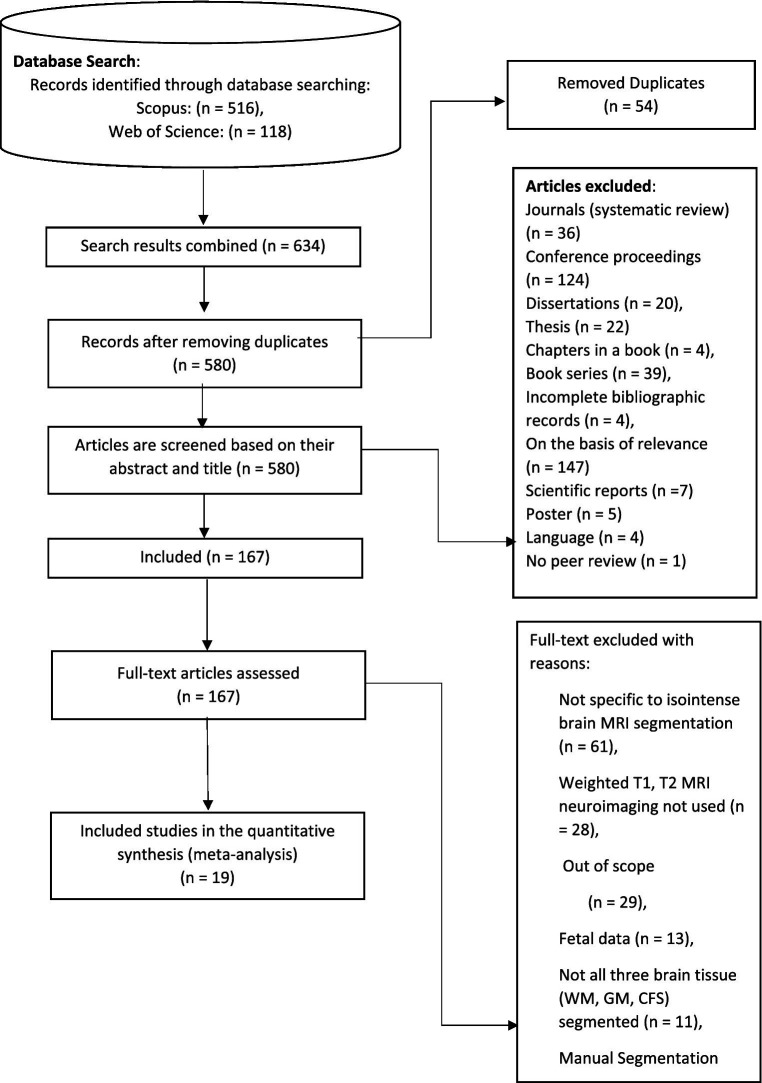
The step of PRISMA for the systematic literature review. Adapted with permission from Liberati et al. (43), licensed under CC BY.

Data abstraction and analysis come last. The remaining publications were assessed, examined, and analysed, and 19 were chosen for in-depth discussion in this paper (see Table 5). Reviews were based on particular studies that addressed the study’s research issue and purpose. Then, by reviewing the article’s title, abstract, and full text, the studies were extracted to find pertinent themes for the current study. Figure 2 depicts a synopsis of the SLR procedure. In this study, quality assessment was based on the checklist suggested and provided by Kitchenham et al. (49) as cited by Usman et al. (50). A three-point scale was used in this study which is Yes/ NO/ Partial. Yes (Y), represented 1, Partial represented 0.5, and NO represented 0, This study used first quartile as the cut-off point which is 3.25. If a study scored less than 3.25 it would be removed from the primary studies.

**Table 5:** Summary of the 19 selected studies using PRISMA approach for isointense brain tissue segmentation.

Authors	Techniques	Modality	Infantile	Development stage at scan	Early-Adult
				Isointense	
(15)	-	T1, T2		✓	
(20)	K- Nearest Neighbour	T1, T2	✓	✓	
(5)	Multi-Atlas	T1, T2, FA	✓	✓	✓
(18)	Random Forest	T1, T2, FA	✓		
(27)	2D CNN	T1, T2, FA		✓	
(25)	SVM	T1, T2		✓	
(51)	Random Forest	T1, T2		✓	
(2)	3D CNN	T1, T2		✓	
(21)	3D CNN	T1, T2		✓	
(24)	3D CNN	T1, T2		✓	✓
(34)	3D CNN	T1, T2		✓	
(6)	FCN	T1, T2		✓	
(52)	3D CNN	T1, T2		✓	
(42)	3D CNN	T1, T2		✓	
(53)	CNN	T1, T2	✓	✓	
(54)	2D CNN	T1, T2		✓	
(28)	3D FCN	T1, T2		✓	✓
(55)	3D CNN	T1, T2		✓	✓
(56)	GAN	T1, T2		✓	✓

The scoring process was Y = 1, P = 0.5, N = 0.

## 4 Results

Below, you will find a review of these 19 studies, with four categories of the methodologies that were examined. Knowledge-driven segmentation methods are covered in the first section. These methods are based on the use of advanced knowledge of brain morphology, including information on the relative position, connection, and structure of brain tissue. The second section presents an approach atlas-based and patch-driven approach. Methods that primarily rely on propagated atlas labels, registration techniques for the best atlas alignment, and various label fusion techniques for multi-atlas methods are all examples of atlas-based approaches. The third section presents machine learning methods such as random forest, k-nearest (*k*NN) neighbour and support vector machine (SVM). When a multi-class classifier is used to create a brain tissue probability map for each tissue type (i.e., WM, GM, CSF), these supervised algorithms are intrinsically well suited for multi-class challenges. Convolution neural network-based deep learning techniques are covered in the final section. In a variety of computer vision applications, including the segmentation of infant brain MRI, CNN has displayed exceptional achievements (42, 57).

### 4.1 Knowledge-based approach

**Figure 3:**
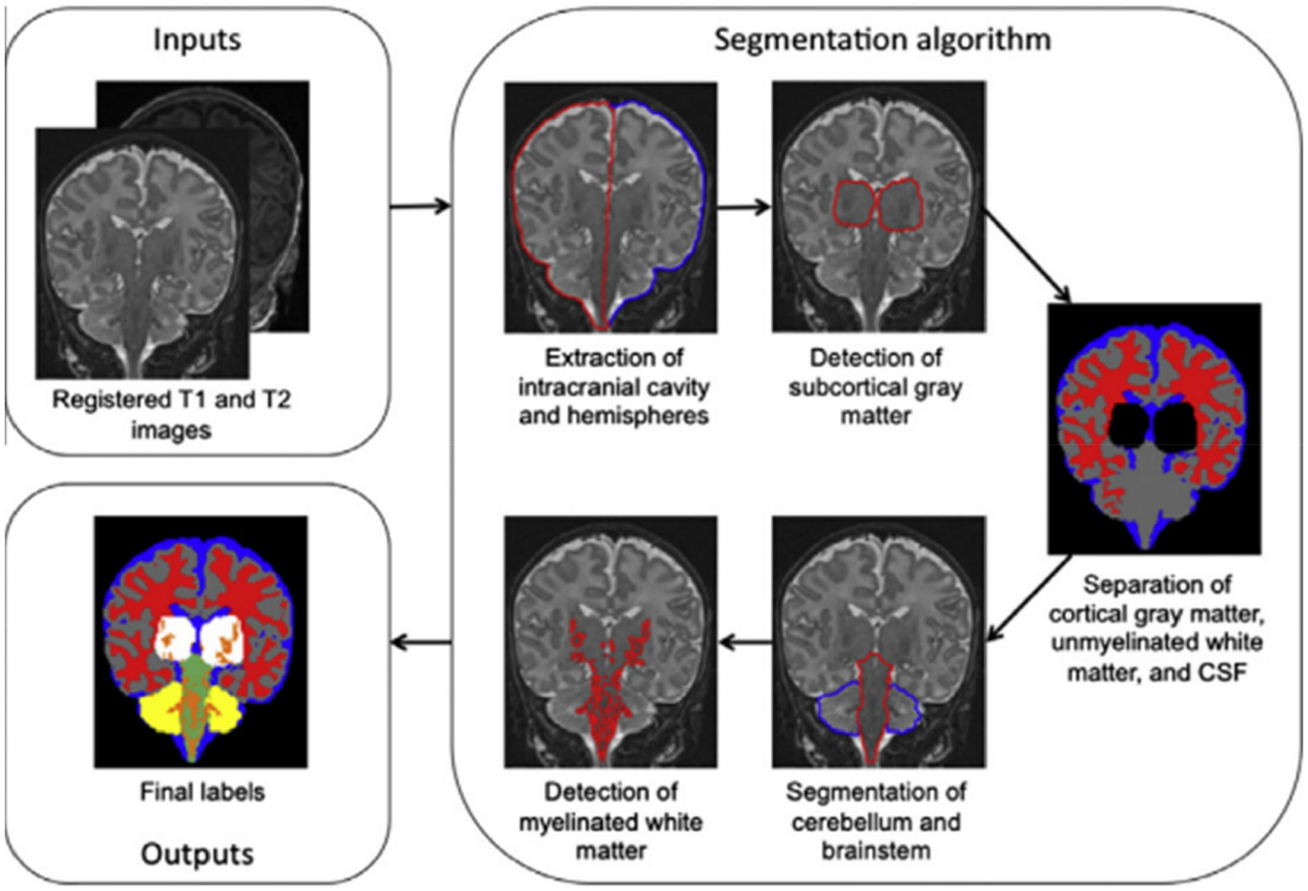
Outline of the segmentation algorithm. Reprinted with permission from Elsevier, Copyright © 2012 Elsevier (15).

By incorporating knowledge of tissue connectivity, structure, and relative placements (15), offer a brain MRI segmentation technique that is based on general and widely acknowledged knowledge of neonatal brain morphology. The authors, for instance, utilised knowledge that the extra-ventricular CSF surrounds the cerebral gray matter, which is itself surrounded by the cortical white matter. The outline in Figure 3 summarizes the segmentation algorithm’s five steps. The procedures involve removing the brain’s intracranial cavity and hemispheres, detecting subcortical gray matter, separating cortical gray matter, unmyelinated white matter, and CSF, segmenting the cerebellum and brain stem, and detecting unmyelinated white matter (15). An infant’s brain’s T1 and T2 MR scans served as the algorithm’s input data.

### 4.2 Atlas-based and patch-driven approach

**Figure 4:**
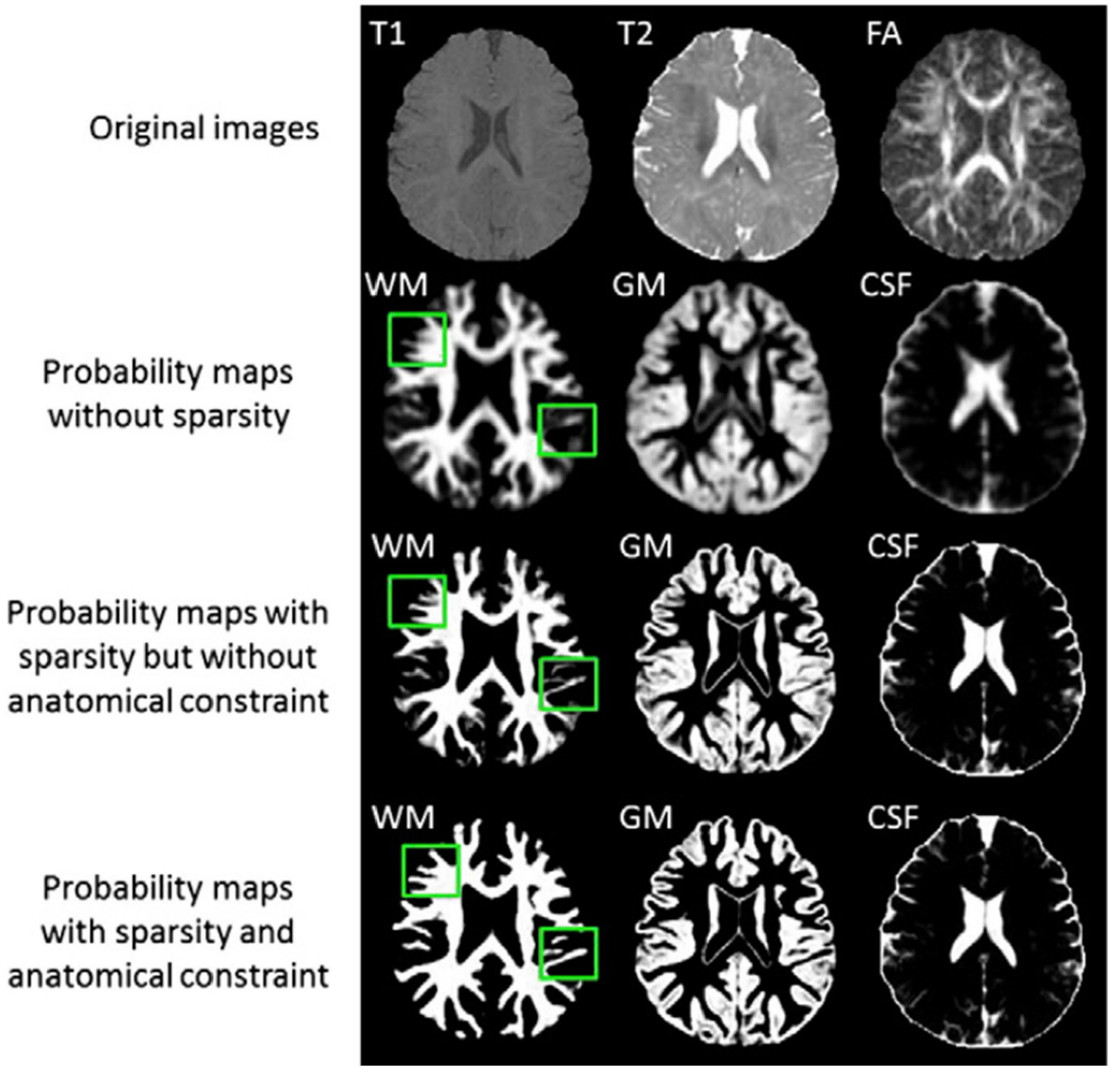
Tissue probability maps calculated using the suggested approach without and with the anatomical restriction, as well as with and without the sparse constraint. Reprinted with permission from Elsevier, Copyright © 2014 Elsevier (5).

The authors provide a basic framework for isointense new-born brain MRI segmentation that uses sparse representation to combine the information from multiple imaging modalities (5). The authors initially create a library made up of a collection of multi-modality images from the training subjects and the ground-truth segmentations that match to those images. T1 and T2 images as well as fractional anisotropy (FA) images make up multi-modality. The training library patches provide a sparse representation of each patch needed to segment a brain image. The generated sparse coefficients are then used to obtain the first segmentation. The initial segmentation will be further considered in light of the patch similarities between the segmented testing picture and the manual segmentation (ground-truth) in the library images in order to enforce the anatomical correctness of the segmentation (5). Figure 4 illustrates the tissue probability maps calculated using the suggested approach.

### 4.3 Machine learning approaches

A segmentation technique based on supervised pixel categorization is suggested by Anbeek et al. (20). Both spatial and intensity characteristics were provided for each voxel. Each brain voxel was classified into one of the eight tissue classes using the k-nearest neighbour (kNN) classifier based on these characteristics. A preterm cohort of 108 infants’ T1- and T2-weighted MR images were obtained at term equivalent age. The brainstem, cerebellum, cortical and central grey matter, unmyelinated and myelinated white matter, cerebrospinal fluid in the ventricles and in the extra cerebral space were all segmented into eight classes using an automatic probabilistic segmentation method. Using leave-one-out tests on seven photos for which a reference standard had been manually established by a subject matter expert, the approach was trained and evaluated (20). The approach was then used on the remaining 101 scans, and the segmentations that resulted were assessed visually by three specialists. The volumes of the eight groups of segmented tissue were then calculated for each subject (20).

**Figure 5:**
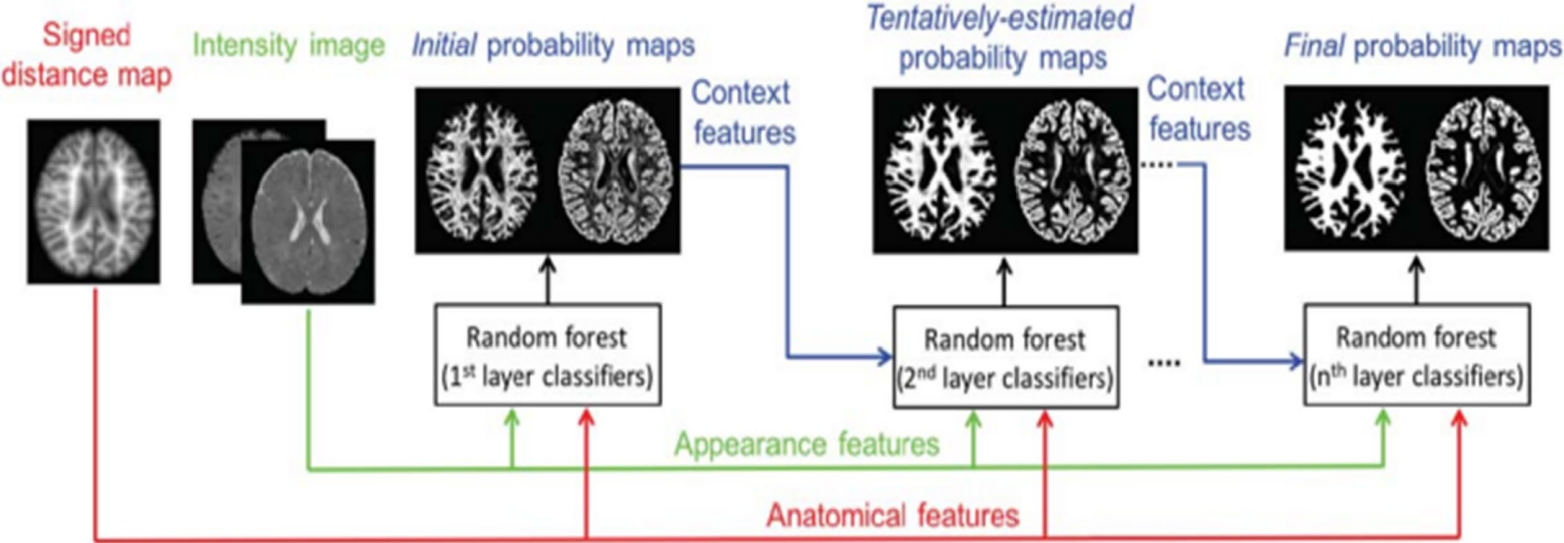
Training flowchart for a series of classifiers for WM versus GM. Reprinted with permission from Wiley, Copyright © 2018 Wiley (51).

A strategy based on learning, employing random forest classifier for infant brain MRI segmentation is proposed by Sanroma et al. (25), Wang et al. (51), and Wang et al. (18). The authors propose a novel learning-based multi-source integration architecture for segmentation (18), where the tissue segmentation challenge is formulated as a tissue categorization challenge. In particular, tissue probability maps for each tissue type can be produced via voxel-wise classification using the random forest classifier, which is naturally suited for multi-class situations. In order to completely capture both local and contextual picture information, a large amount of training data with high data dimensions can be handled by random forest. This allows for the exploration of a huge number of image features. Additionally, an anatomy-guided tissue segmentation for 6-month-old new-born brain MRIs with autism risk was presented by Wang et al. (51). Intensity images’ 3D Harr-like feature extract is input to a random forest classifier, which outputs a class classification. Figure 5 shows a training flowchart for a series of classifiers for WM versus GM. A combination of strategies is presented by Sanroma et al. (25) for infant brain MRI segmentation. The standard approaches include support vector machine (SVM) and multi-atlas joint label fusion, which serve as examples of registration-based methods. A collection of several annotated photos is necessary for both registration and learning-based approaches.

### 4.4 Deep learning methods

As of late, deep learning techniques centred around convolutional neural networks (CNNs) have demonstrated exceptional execution around a range of computer visualization and photograph evaluation usages in the clinical space (30, 31, 35, 36, 39). Convolutional neural networks were used in the majority of the publications found through the systematic literature review study using the PRISMA approach; 12 out of the 19 articles used CNNs.

#### 4.4.1 Deep fully convolutional neural networks

**Figure 6:**
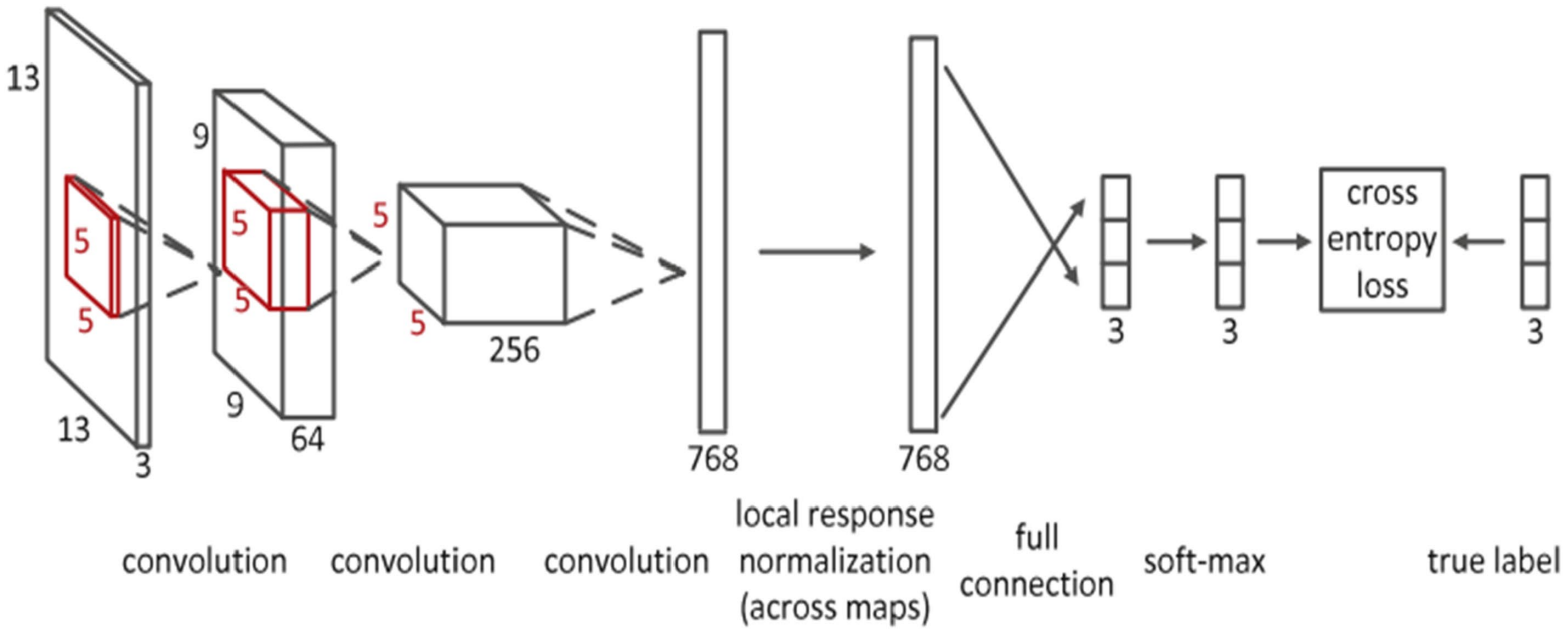
Convolutional neural network’s detailed architecture using inputs in patches that are 13 by 13 in size. Reprinted with permission from Elsevier, Copyright © 2015 Elsevier (27).

Deep convolutional neural networks (CNN) are suggested for multi-modality MRI segmentation of isointense brain tissue (27). According to Figure 6, the authors created CNN architectures with three input feature maps for 13 × 13 T1, T2, and FA image patches. There are three convolutional layers and one fully connected layer used. Local response normalization and softmax layers were also used in this network.

It is advised that more research be done on deep convolutional neural networks and suggestive annotations for new-born brain MRI segmentation (42). This study uses an ensemble of semi-dense fully convolution neural networks with T1- and T2-weighted MRI as the input to examine the issue. The study shows that there is a strong correlation between segmentation mistakes and ensemble agreement. The approach thus offers measurements that can direct local user corrections. The performance of deep architectures was also examined by the authors in relation to the effects that early or late fusion of various image modalities might have (42).

**Figure 7:**
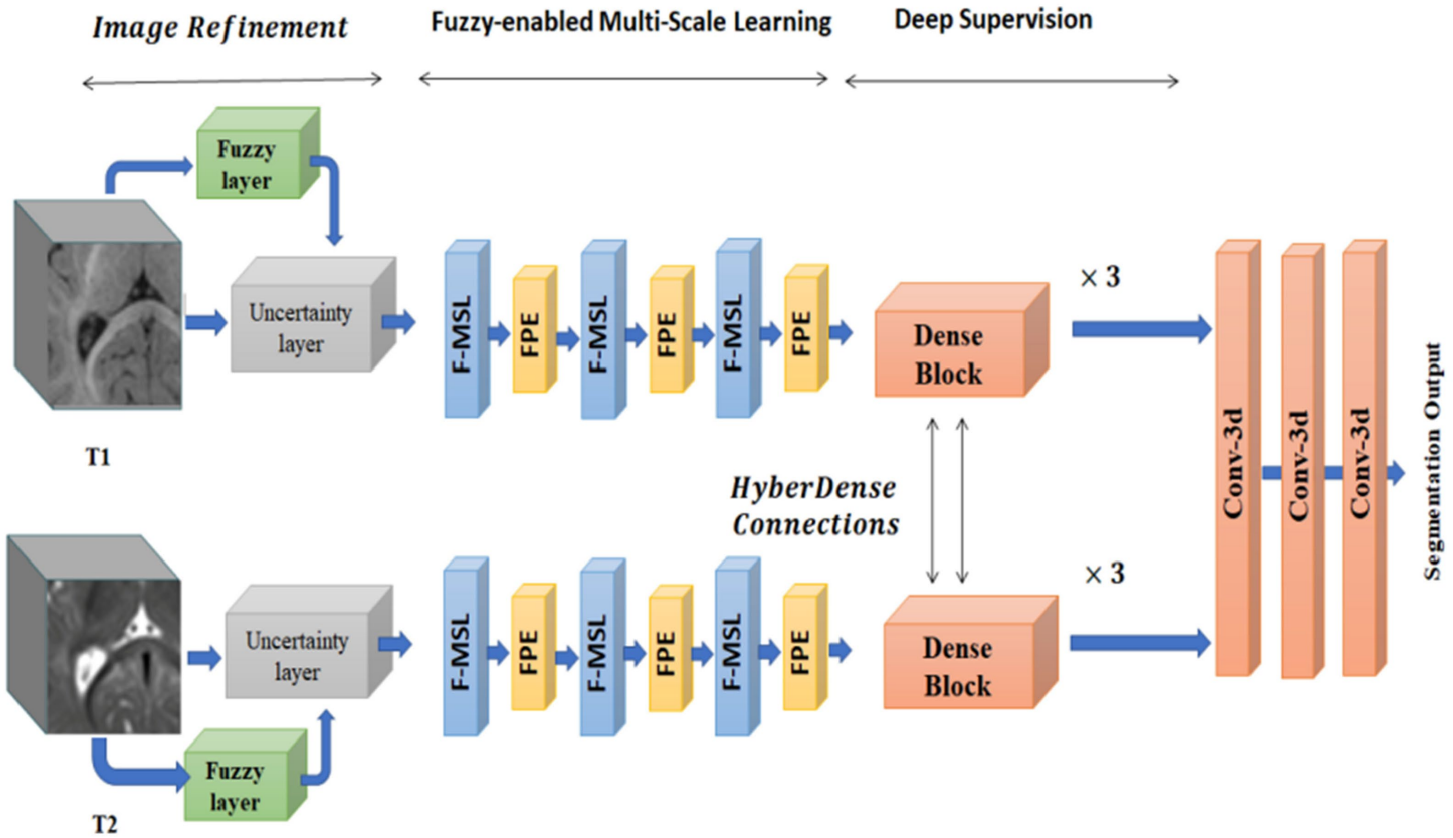
The structure of the fuzzy-guided framework that has been presented for multimodal brain MRI segmentation. Reprinted with permission from IEEE, Copyright © 2022 IEEE (55).

A fuzzy-informed deep learning segmentation guided network by pertinent principles, as well as building blocks to learn multimodal information from MRI images, are also proposed by Ding et al. (55). Figure 7 shows the architecture, which consists of three primary processing steps: deep supervision, fuzzy-enabled multi-scale learning, and image refinement. A volumetric fuzzy pooling layer applies fuzzification, accumulation, and de-fuzzification to the neighbourhoods of adjacency feature maps to mimic the local fuzziness of the volumetric convolutional maps. To enable the extraction of brain characteristics in various receptive fields, the fuzzy-enabled multiscale feature learning module is designed using the VFP layer. A fuzzified multichannel dense model for multimodal segmentation has also been introduced.

A powerful 2D convolutional network called Rubik-Net uses the bottleneck structure and residual connections to improve information transfer while requiring fewer network parameters. On the iSeg2017, iSeg2019, and BrainWeb datasets, the Rubik-Net demonstrated good results in terms of segmentation accuracy (54).

#### 4.4.2 Hyper densely connected CNNs

**Figure 8:**
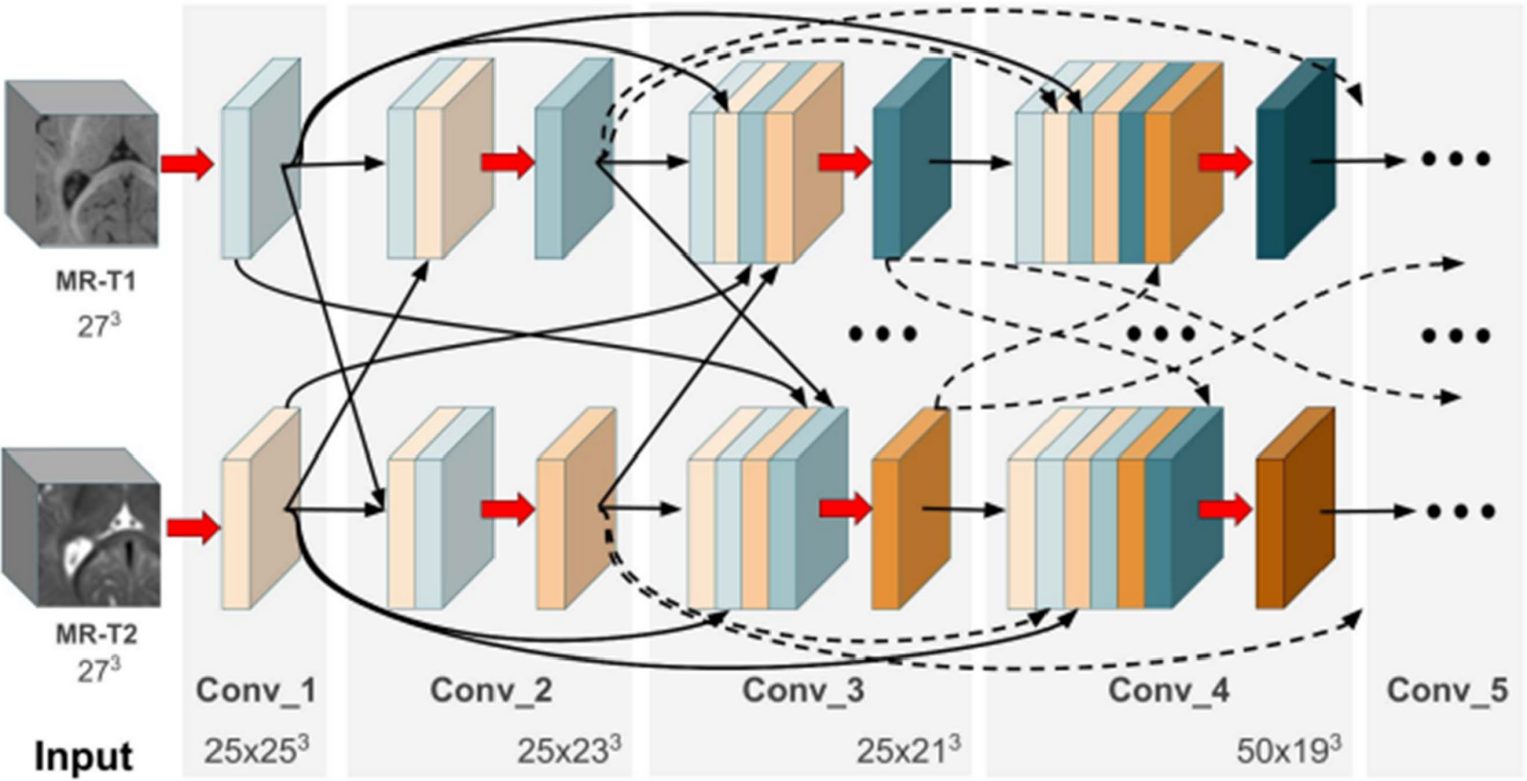
In the case of two picture modalities, a portion of the proposed HyperDenseNet. Each area of gray stands for a convolutional block. Black arrows denote dense connections between feature maps, while red arrows represent convolutions. Reprinted with permission from IEEE, Copyright © 2019 IEEE (2).

Hyper-densely connected CNNs have been employed by Basnet et al. (53), Bui et al. (21), Dolz et al. (2), Hashemi et al. (34), and Qamar et al. (24) in isointense infant brain MRI segmentation. The idea of dense connection is extended to multi-modal segmentation problems by a 3D fully convolution neural network developed by Dolz et al. (2). Each image modality has a path, and dense connections can be shown in Figure 8 for both airings of layers that are on the same path as one another as well as layers that are on distinct paths.

**Figure 9:**
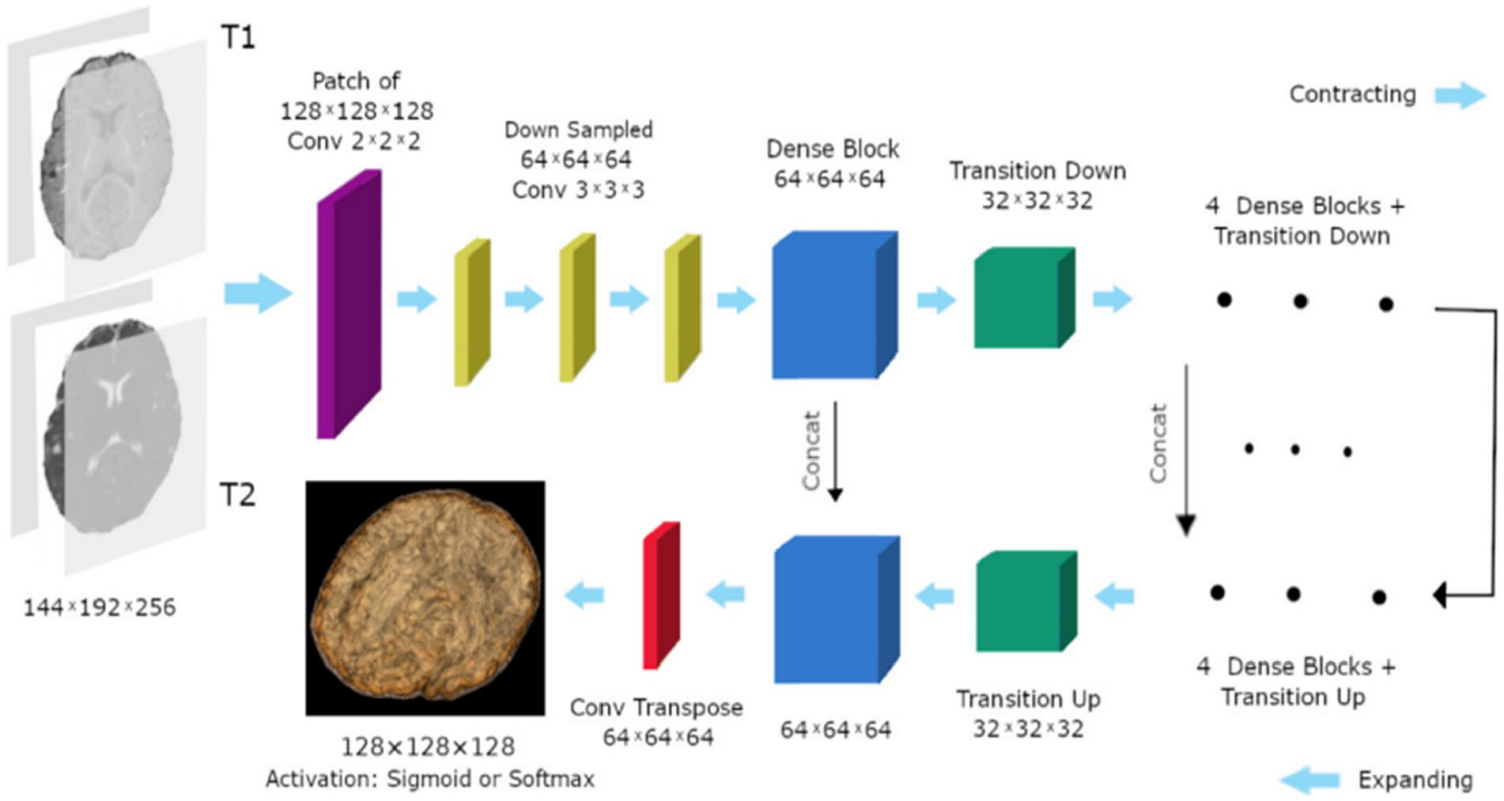
The study’s 3D FC-DenseNet architecture uses a 222 convolution with stride 2 (purple) to downscale the input patch from 128 × 128 × 128 to 64 × 64 × 64 in the first layer. The patch is upsampled from 64 × 64 × 64 to 128 × 128 × 128 using a 222 convolution transpose with stride 2 (red) before the activation layer. With the help of this deep architecture, we were able to overcome memory size restrictions with big input patches, retain a wide field of vision, and add five skip connections to enhance the flow of local and global feature data. Reprinted with permission from, licensed under CC BY-4.0 (34).

A deep densely connected network called 3D FC-DenseNet has been suggested by Hashemi et al. (34). Due to its early downsampling and late upsampling layers, the network in Figure 9 has eight times the usual patch sizes (128 × 128 × 128 vs. 64 × 64 × 64), more depth, skip connections, and parameters than its predecessors.

“Deeper is the better” concepts plays an important role in deep learning architecture (24). A hyper-densely connected convolution neural networks for segmentation of infant brain MRI is presented by Qamar et al. (24). The suggested model offers close connections between layers to enhance the network’s flow information performance. The algorithm employs T1 and T2 as input. On the other hand (21), carefully designed a fully convolutional densely connected network with skip connections, allowing for the direct combination of data from various densities of dense blocks to produce extremely precise segmentation results.

#### 4.4.3 Generative adversarial networks

A network known as a “generative adversarial network” (GAN) is made up of two networks: a generator (G) that creates a false image from a noise vector and a discriminator (D) that determines the difference between produced and real data (56). It is advised to use a multi-stage Generative Adversarial Network for image segmentation (56). The model creates a rough contour of the background and brain tissues in the first stage. The model then creates a more detailed contour for the white matter, gray matter, and cerebrospinal fluid in the subsequent stage. The performed fusion of the *coarse* and *refined* outliners.

#### 4.4.4 UNet architecture

**Figure 10:**
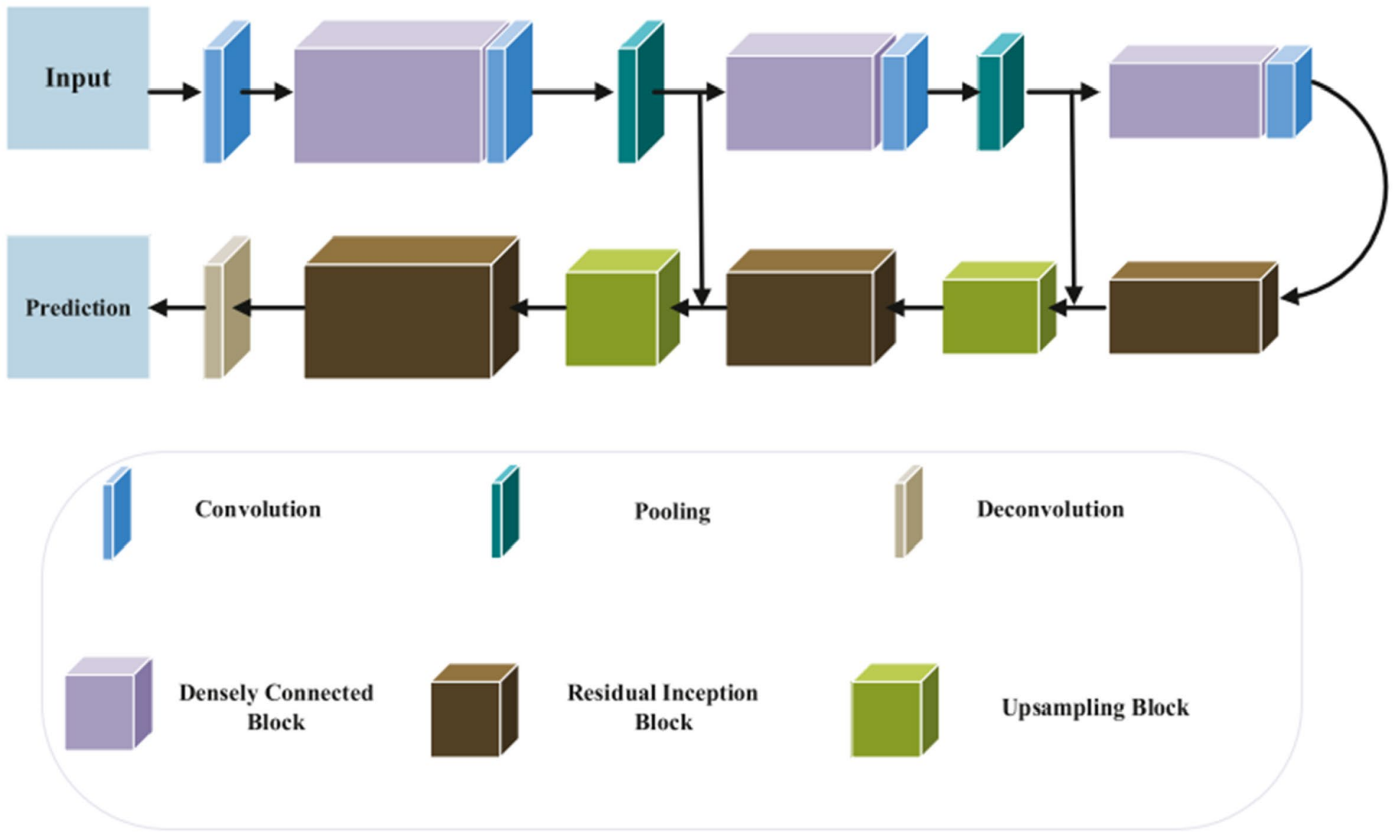
Segmentation of 3D MRI brain images using a suggested network design. In the suggested approach, DenseNet and Inception-ResNet are used concurrently. Reprinted with permission from Elsevier, Copyright © 2020 Elsevier (52).

The UNet model is one of the most popular convolution neural networks (CNN) that have been successfully used to medical imaging tasks (38, 52, 53). Convolutional, pooling, and up-sampling layers make up the UNet model (52). An architecture for segmenting the baby brain is shown in Figure 10. The network has two paths: a downsampling encoder path and an upsampling decoder path. Reduced feature map resolution and increased receptive field are the goals of downsampling in the encoder path. The residual inception and upsampling blocks make up the up-sampling procedure in the decoder pipeline. Particularly, local features are found in the shallower layers, whereas global features are found in the deeper layers. For new-born brain segmentation, the concatenation of the several levels of upsampling feature maps enables the capture of multiple contextual information. To classify the concatenated features into the target classes (WG, GM, CSF), a classifier is made up of a Conv (1 × 1 × 1). The brain probability maps that were produced using the Softmax classifier (52).

**Figure 11:**
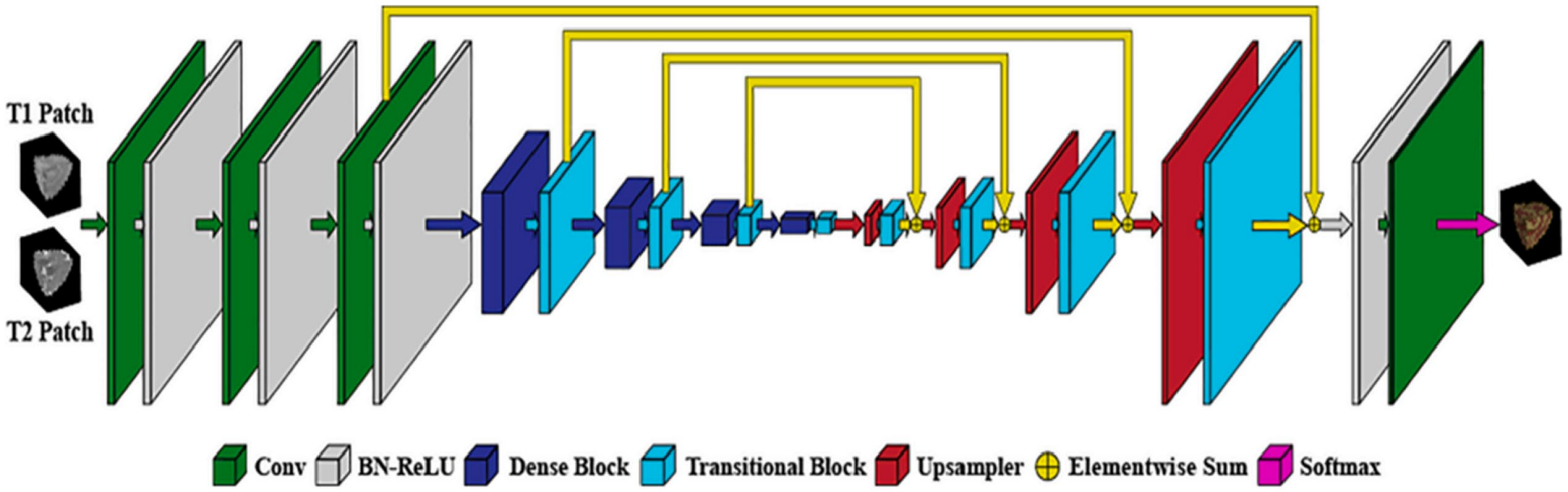
Architecture of the proposed network. Reprinted with permission from Elsevier, Copyright © 2021 Elsevier (53).

On the other hand (53), proposed In order to partition the brain tissues into the three categories of white matter, gray matter, and cerebrospinal fluid, a novel 3D CNN architecture that is based on the U-Net structure is described. The basic idea behind the proposed method is to use residual skip-connections and densely connected convolutional layers, as shown in Figure 11, to reduce the number of parameters in the network, improve gradient flow, and increase representation capacity. In addition, the suggested network is trained using the loss functions, cross-entropy, dice similarity, and a combination of the two.

In addition, Triple Residual Multiscale Fully Convolutional Network, a deep network design based on U-Net, is suggested by Chen et al. (6). The model is composed of encoder and decoder process. Encoder procedure comprises: tradition 2D convolution, max-pooling and residual block while the decoder procedure comprises deconvolution, residuals multiscale block, concatenate block and traditional 2D convolution. Furthermore, APR-Net, a new 3D fully convolutional neural network for segmenting brain tissue, is presented by Zhuang et al. (28). The model is made up of several encoded streams and one decoded stream, three primary components make up APRNet: Multi-modal cross-dimension attention modules, 3D anisotropic pyramidal convolutional reversible residual sequence modules, and the core of the APRNet.

The common evaluation metrics that were applied to the 19 studies that were obtained for this analysis utilizing the PRISMA approach are detailed in the section that follows.

## 5 Evaluation metrics

To assess the accurateness of an automatic segmentation algorithm: Dice Similarity Coefficient (DSC) (58, 59), Modified Hausdorff distance (MHD), where the 95-th percentile of all Euclidean distance is utilized, along with Average Surface Distance (ASD). The initial method computes intensity of overlap amongst the segmented area together with the ground truth, while the additional two techniques estimate the border distances (2, 21).

19 out of 21 of the articles obtained from the PRISMA approach employed one or more of the evaluation metrics (DSC, MHD, and ASD). Table 6 presents a list of all 19 studies and the metrics applied to assess the results of an segmentation algorithm.

**Table 6:** A list of evaluation metrics employed by the 19 selected articles using PRISMA approach.

Authors	Evaluation Metrics	Dataset	DSC	WM			GM			CSF	
				MHD	ASD	DSC	MHD	ASD	DSC	MHD	ASD
(15)	Dice		0.94			0.92			0.84		
(20)	Dice		0.47			0.91			0.75		
(5)	Dice		0.89			0.87					
(18)	Dice, MHD	NeoBrain12	0.86			0.88			0.92		
(27)	Dice, MHD		0.86	0.28		0.85	0.24		0.83	0.44	
(25)	Dice	iSeg2017	0.97			0.90			0.95		
(51)	Dice, MHD	NDAR	0.89	0.28		0.90	0.24		0.92	0.43	
(2)	Dice, MHD	iSeg2017, MRBrainS13	0.89	1.78	6.03	0.86	1.34	6.19	0.83	2.26	7.31
(21)	Dice, MHD, ASD	iSeg2017	0.91	5.92	0.39	0.91	5.75	0.34	0.94	13.64	0.13
(24)	Dice, MHD, ASD	iSeg2017	0.90	6.88	0.39	0.92	5.63	0.31	0.96	9.00	0.11
(34)	Dice, MHD, ASD	iSeg2017	0.90	7.1	0.36	0.92	9.55	0.31	0.96	8.85	0.11
(6)		iSeg2017									
(52)	Dice, MHD, ASD	iSeg2017	0.91	6.56	0.37	0.92	5.75	0.31	0.96	9.23	0.13
(42)	Dice, MHD, ASD	iSeg2017	0.90	7.45	0.41	0.92	6.06	0.34	0.96	9.13	0.12
(53)	Dice, MHD, ASD	iSeg2017, IBSR18	0.90	6.77	0.39	0.91	5.94	0.32	0.95	9.20	0.11
(54)	Dice, MHD, ASD	iSeg2017, iSeg2019, IBSR, BrainWeb	0.86	8.92	0.53	0.81	8.17	0.53	0.82	11.6	0.53
(28)	Dice, MHD, ASD	iSeg2017, MRBrainS13	0.91	6.22	0.35	0.92	6.41	0.32	0.95	9.13	0.12
(55)	Dice, MHD, ASD	iSeg2017	0.92	6.21	0.29	0.93	5.24	0.28	0.96	7.66	0.09
(56)	Dice	iSeg2017, MRBrainS13	0.88			0.93			0.93		

In addition, Dice Similarity Coefficient, Modified Hausdorff Distance, Average Surface Distance metrics were also employed by iSeg-2017 organizers to assess the accurateness of the contesting segmentation techniques (16, 17):

To measure the intersection amongst separations, outcome X together with ground truth Y, the Dice Similarity Coefficient is characterised as tails:


(1) $$DSC=\frac{2\left|X\cap Y\right|}{\left|X\right|+\left|Y\right|}$$

where *X* and *Y* represent two segmentation labels created physically and computationally, correspondingly, |*X*| represents the amount of optimistic portions in the binary segmentation *X*, and |X∩Y| is the amount of common optimistic elements by *X* together with Y. A bigger DICE reveals a greater intersection among the physical and projecting division regions. The threshold should not be greater than 1 (16, 17).

Allow *R* along with *S* be the series of voxels within the physical and predicative segmentation limit, correspondingly. A modified Hausdorff distance (MHD) is described as follows:


(2) MHD(R,S)=max{h(R,S),h(S,R)}

where $h\left(R,S\right)=\frac{1}{N_{c}}\underset{r\in r}{\sum }d\left(r,S\right)$ and d(r,S)=∥r∈Rminr−s∥ with ∥.∥ representing the Euclidean distance. A lesser MHD coefficient implies bigger resemblance between manual and predictive segmentation contours (7, 60). The maximum MDH from set X to set Y is a max function defined as 95%.

The third computation metric is the average surface distance (ASD), termed as:


(3) $$ASD\left(C,D\right)=\frac{1}{2}\left(\frac{\sum_{\in ivS_{C}}\left\|v_{i}-v_{j}\right\|\in jvS_{D}\min}{\sum_{v_{i}}\in S_{C}\;1}+\frac{\sum_{\in jvS_{D}}\left\|v_{i}-v_{j}\right\|\in ivS_{C}\min}{\sum_{v_{j}}\in S_{D}\;1}\;\right)$$

where $S_{C}$ and $S_{D}$ signify the outside meshes of C and D, correspondingly. A lesser ASD number implies superior segmentation accuracy (17).

The performance comparison of this study was done using DCS, MHD and ASD, comparing it with previous studies (21, 24, 34, 42, 52). This shows the room of improvement or lack of improvement of our study using different evaluation metrics. The evaluation metric employed are DSC, MHD, and ASD for white matter (WM). The most favourable results of DCS was which was highest was 0.97, achieved by Sanroma et al. (25) followed by Gui et al. (15) which obtained DSC value of 0.94. Other authors have results less than 0.94. Regarding MHD results, the most optimal results were obtained by Luan et al. (54), which identified a value of 8.92 (11) followed and obtained the results of 6.03.

In addition to that; DSC, MHD, and ASD were computed to identify gray Matter (GM). The most accuracy results were obtained for DSC are 0.93 (55, 56), MHD of 9.55 was obtained by Hashemi et al. (34). For ASD (11), achieved a value of 6.19. Furthermore, CSF accuracy was measured using DSC, MHD, and ASD. Pertaining DCS, the most favourable accuracy was 0.96 supported by Hashemi et al. (34), Qamar et al. (24), Qamar et al. (52), Dolz et al. (42), and Ding et al. (55). The most accuracy value of the metric MHD was 13.64 which was supported by Bui et al. (21). The most favourable metric value for ASD was 7.31 which was supported by Dolz et al. (11).

The most promising algorithm is supported by Dolz et al. (11). Their study was produced most accuracy when using WM, GM, and CSF. Interestingly, no strategy had a statistically significant superior performance than all other methods for segmentation of WM, GM, and CSF across any parameter. For example (25), obtained the highest median in terms of DCS for white matter (WM). Nonetheless, the differences between their findings and those of (15) are not statistically significant. Furthermore, Dolz et al. (11) has the highest ASD values for both WM, GM, and CSF, but one of the lowest MDH medians for WM, GM, and CSF. As a result, there is no discernible, statistically significant difference with any other methods.

The following dataset were used by in the 19 studies selected using the PRISMA.

iSeg-2017 dataset is a publicly available to the research community^1^ consisting of 10 infant subjects (5 females and 5 male) with manual labels were provided for training and 13 infant subjects (7 females and 6 male) were provided for testing. However, manual labels for testing subjects are not provided (16). In addition, iSeg-2019 challenge was done with the aim of promoting automatic segmentation algorithms on infant brain MRI from multiple sites, MR images from four different sites as training, validation, and testing datasets, respectively are available from https://iseg2019.web.unc.edu/.

Three separate image sets of premature babies are included in the NeoBrainS12 data set: (i) axial scans taken at 40 weeks corrected gestational age; (ii) coronal scans taken at 30 weeks corrected gestational age; and (iii) coronal scans taken at 40 weeks corrected gestational age. At the neonatal critical care unit of the University Medical Center Utrecht in the Netherlands, all scans were performed as part of routine clinical procedures. You can get the remaining photos from the first two sets along with the appropriate manual annotations from the NeoBrainS12 website at http://www.miccai2012.org and use them as training data (61).

MRBrainS13 challenge workshop at the Medical Image Computing and Computer Assisted Intervention (MICCAI) conference provided dataset consisting of 20 subjects (mean age ± SD = 71 ± 4 years, 10 males, 10 female) were selected from an ongoing Computational Intelligence and Neuroscience 3 cohort study of older (65–80 years of age) functionally independent individuals without a history of invalidating stroke or other brain diseases. This dataset is publicly available from http://www.miccai2013.org (62).

Along with magnetic resonance brain image data, the Internet Brain Segmentation Repository (IBSR) offers manually guided expert segmentation results. Its goal is to promote the analysis and advancement of segmentation techniques https://www.nitrc.org/projects/ibsr.

Through data sharing, data harmonization, and the publication of study findings, the National Database for Autism study (NDAR), a research data repository supported by the National Institutes of Health (NIH), seeks to further the understanding of autism spectrum disorders (ASD). In addition, NDAR acts as a platform for the scientific community and a gateway to numerous additional research repositories, enabling data aggregation and secondary analysis. Dataset can be accessed from https://www.re3data.org/repository/r3d100010717

## 6 Findings and limitation of the presented frameworks

The findings of this study and drawback of the concerned frameworks on isointense brain MRI segmentation can be seen in Table 6.

### 6.1 Findings

Deep learning methods are popular in isointense brain MRI segmentation, specifically convolution neural networks. An interesting discovery is that 13 of the 19 studies obtained using PRISMA employed convolution neural networks. In addition, Dice similarity coefficient (DCS) was the most frequently used evaluation metrics, where 17 out of the 19 studies used DCS. Modified Hausdorff Distance (MHD) was also employed in 13 studies out of 19, while Average Surface Distance (ASD) was the least utilized evaluation metrics, where nine studies out of the 19 used it. Furthermore, the most commonly used dataset for training and testing was from MICCAI iSEG-2017 Grand Challenge on 6-month infant brain MRI segmentation as illustrated in Table 6. iSEG-2017 dataset is a publicly available to the research community^2^ consisting of 10 infant subjects (5 females and 5 male) with manual labels were provided for training and 13 infant subjects (7 females and 6 male) were provided for testing. However, manual labels for testing subjects are not provided.

### 6.2 Limitation of the presented frameworks

Limitations presented from the assessed frameworks included the omission of ensemble to improve the evaluation metrics. Another studies used Dice similarity coefficient (DCS) and did not compare it with Modified Hausdorff Distance (MHD) and Average Surface Distance (ASD) to provide better results. On the other hand, some authors applied DCS and MHD and did not compare it with ASD to provide better results. Wilcoxon signed-rank test with all-against-all was used to see whether any study performs noticeably better than the others in terms of DCS, MHD, and ASD. Surprisingly, no study was able to partition WM, GM, and CSF across all parameters (DCS, MHD, and ASD) with a substantial statistically significant performance advantage over all other studies. In order to detect the significant difference, ensemble techniques must be employed in conjunction with CNN, and the segmentation error can decrease in order to improve the model. With minimal user interaction, this idea has the potential to deliver expert-level performance.

Most researchers do not focus on improving the accuracy of the model, reducing the amount of Rubik convolutional calculations, and using multi-axis information more efficiently (54). While other avoid image processing due to the lack of datasets (56). Researchers are lacking to integrate different deep fuzzy structures to model data ambiguity and further explore training of deep fuzzy models using incremental and reinforcement learning. In addition, comparison of the research and other study to evaluate performance of proposed architectures using other challenges to take advantage of multi-modal data was lacking in their studies (24). A large amount of researchers have focused on image recognition and classification, there is a lack of CNNs focusing on semantic image segmentation (11). Some emerging research approach such as FCNN minimize redundant convolution results in computation being more efficient. Also few researchers have focused on 3D CNN-ensemble learning strategy used to improve performance (42). To overcome the challenges, single non-linear convolutional can be used. Lastly, this study considered paper published between 1^st^ of January 2012 and 31^st^ of December, 2022.

## 7 Limitation and future work

The limitation of this study come from fact that number of images in iSEG-2017 dataset is not enormous, it consists of only 10 (T1-weighted and T2-weighted MRI) for training and 13 (T1 and T2 MRI) for testing. In addition, the ground truth labels for the test instance are not available. In this study, both T1-weight and T2-weight MRI are studied. In future, only T1-weight or T2-weight MRI will be considered. In addition, accurate segmentation of child brain MRI is extremely difficult than grown-up brain segmentation, because of low tissue differentiate, excessive noise, continuing WM Mylenium, and uncompromising incomplete volume effects which makes tissues to remain miscategorised together with diminishing the exactness of the segmentation algorithm (14, 16, 63).

Most of the CNN models, experiments were performed on computational servers or CPU with a graphic processing unit (GPU) memory. Furthermore, similar article written by same authors were treated as separate paper based on different ideas of contribution (5, 18). Most dataset are already cleaned as secondary dataset, as a result, they contain lots of errors which can be minimized by re-cleaning the dataset. In the future, data augmentation could be applied to possible improve the results, by amplifying the size of the dataset. Furthermore, other evaluation metrics could be utilized such Jaccard index which is also common for the evaluating of image segmentation tasks. The same algorithms selected in this study can be applied to adult brain MRI segmentation.

## 8 Conclusion

This systematic review investigates isointense brain MRI segmentation. An extensive literature search for relevant studies published in the period of 2012 to 2022 and finally identified 19 primary studies that are pertaining to the four research questions (RQs) raised in this review. A summarized research approach of the existing literature along with the research contribution, evaluation metrics, datasets, finding and future recommendations to study isointense brain MRI segmentation models are described. The principle findings of this review are summarized as follows:

- [RQ-1] The detailed review of infant brain MRI segmentation techniques and deep learning techniques has been deliberated in Section 4 and Sub-Section D of Section 4, respectively. The summarized review is examined in Table 5.
- [RQ-2] Section 5 of this study reviews datasets. Table 6 presents the evaluation metrics and the most frequently used dataset for isointense brain MRI segmentation.
- [RQ-3] It has been observed that deep learning techniques are popular in isointense brain MRI segmentation. Thirteen out of the nineteen studies used convolutional neural network and Dice Similarity Coefficient is also the most used evaluation metric from the presented frameworks.
- [RQ-4] Future works and limitations from researcher play a vital role to explore further research in a relevant domain. To answer this RQ, the limitations and future works of deep learning technique and evaluation metrics is discussed in Section 6 and 8, respectively. It was found that most studies recommended the use of data augmentation to amplify the size of the dataset, which could possibly improve the results.

## Data availability statement

The original contributions presented in the study are included in the article/supplementary material, further inquiries can be directed to the corresponding author.

## Author contributions

SM and SV contributed on literature review, SM and SV defined the research problem, SM and SV designed and implemented a framework, SM and SV analysed and computed the results. All authors listed have made a substantial, direct, and intellectual contribution to the work and approved it for publication.

## Footnotes

^1^ http://iseg2017.web.unc.edu

^2^See footnote 1.
